# P-1657. Difference in reactogenicity events between mRNA and Protein-subunit Vaccines: Results from the Booster Epidemiological Evaluation of Health, Illness, and Vaccine Efficacy (BEEHIVE) Study, a U.S. randomized trial of 2023-2024 COVID-19 vaccines (XBB.1.5)

**DOI:** 10.1093/ofid/ofaf695.1832

**Published:** 2026-01-11

**Authors:** Sarang K Yoon, Matthew S Thiese, Andrew Phillips, German L Ellsworth, Sarah W Ball, Elizabeth Rowley, Steph Battan-Wraith, Rebecca Fink, Adam Yates, Seth Toback, Lisa M Dunkle, Matthew D Rousculp, Hongwei Zhao

**Affiliations:** University of Utah School of Medicine, Salt Lake City, UT; University of Utah - Rocky Mountain Center for Occupational and Environmental Health, Salt Lake City, Utah; University of Utah, Salt Lake City, Utah; University of Utah, Salt Lake City, Utah; Westat, Newton, Massachusetts; Westat, Newton, Massachusetts; Westat, Newton, Massachusetts; Westat, Newton, Massachusetts; Westat, Newton, Massachusetts; Novavax, Inc., Gaithersburg, Maryland; Novavax, Inc., Gaithersburg, Maryland; Novavax, Inc., Gaithersburg, Maryland; University of Utah, Salt Lake City, Utah

## Abstract

**Background:**

Vaccine reactogenicity, defined as local and systemic reactions, is a leading factor for COVID-19 vaccine hesitancy in adults^1-4^. As the perceived risk of COVID-19 decreases, individuals may become less permissive towards vaccine-associated adverse reactions.

**Figure 1.**
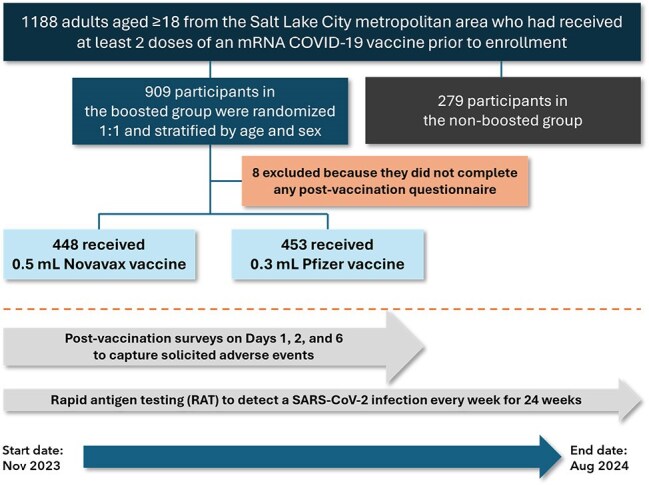
Study Design

**Figure 2.**
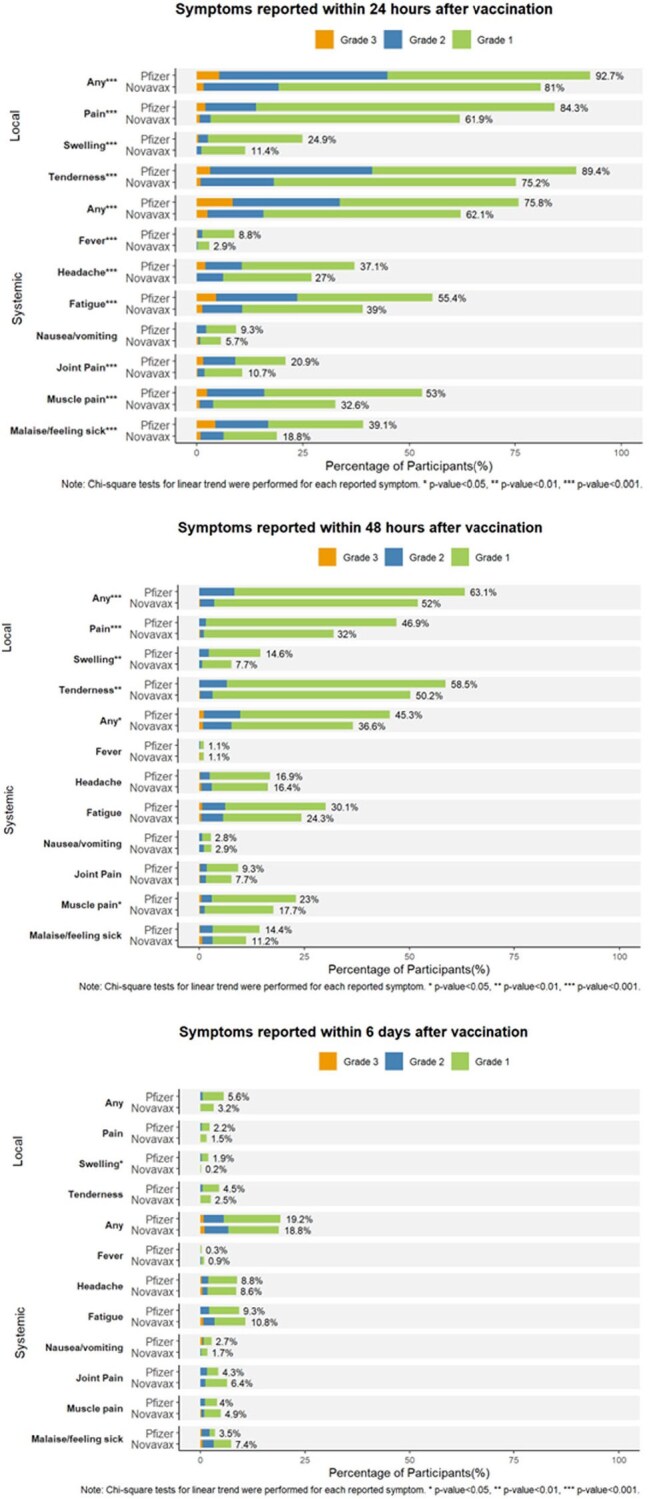
Local and systemic reactogenicity events after Novavax or Pfizer vaccines within 1, 2, and 6 days

**Methods:**

This double-blinded RCT compared two 2023-2024 (XBB.1.5) vaccines Novavax (NVX), a protein-based vaccine, and Pfizer-BioNTech (mRNA) over a 24-week period between Nov 2023 and Aug 2024 (Fig 1). Solicited reactogenicity symptoms (systemic and local), were evaluated using a post-vaccination questionnaire at 3-time intervals (1-, 2-, and 6-days post-vaccination). Reactogenicity was assessed for occurrence and maximum intensity (according to FDA toxicity grade). Tests for linear trend were performed.

**Figure 3.**
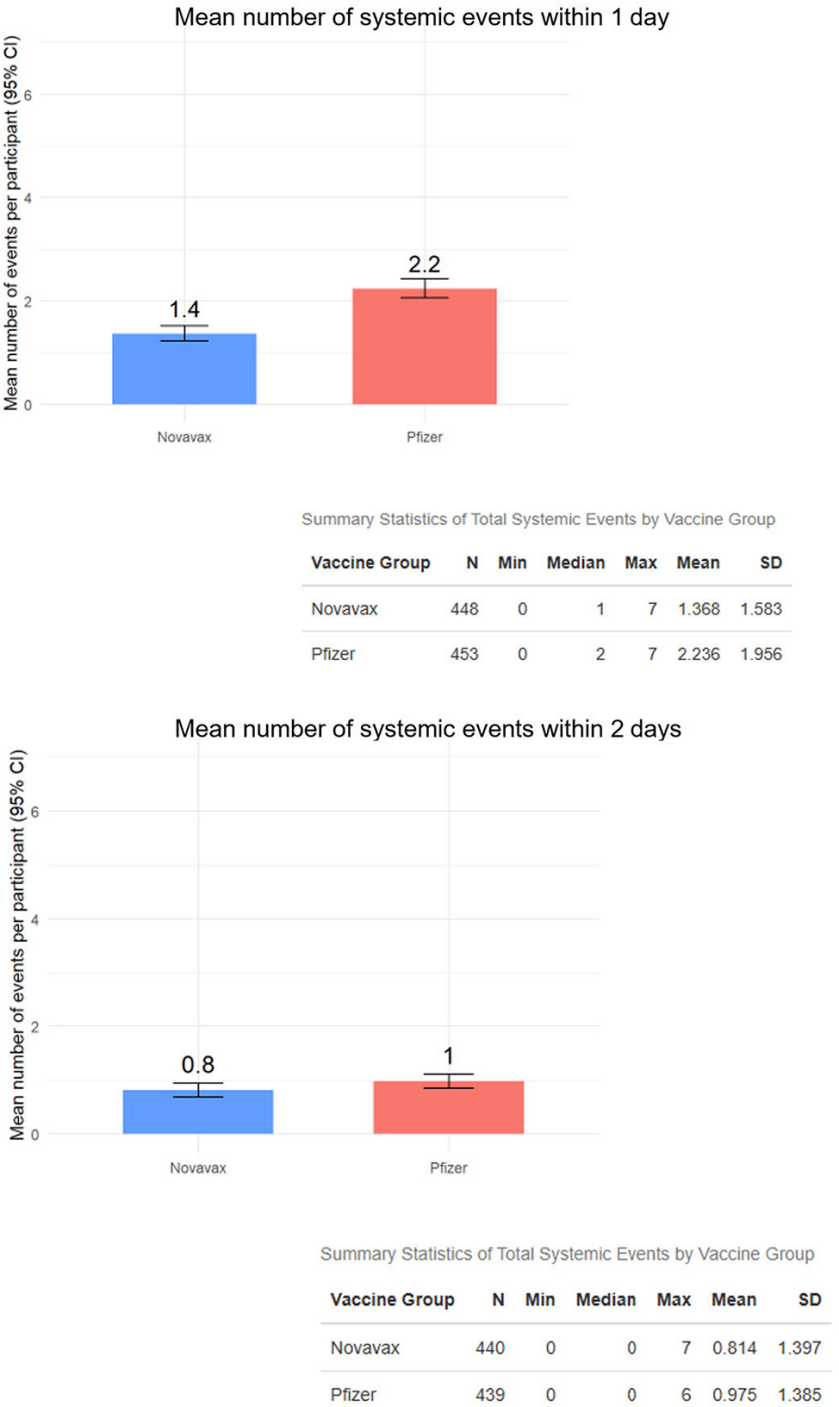
Summary of solicited systemic reactogenicity events after Novavax or Pfizer vaccines within 1 and 2 days post-vaccination

**Results:**

901 participants [mean age (SD): 40.5 (15.95); 55% female; White (91%); Hispanic (11%)] completed at least one of the questionnaires (NVX n=448; mRNA n=453); all previously received ≥2 doses of mRNA vaccines prior to enrollment with 89% reported receipt of ≥3 COVID-19 vaccinations and 60% reported a prior SARS-CoV-2 infection.

In review of the solicited systemic reactogenicity during the first day post-vaccination, 62.1% of NVX recipients reported experiencing any event versus 75.8% of mRNA recipients (p< 0.05) (Fig 2). On average, NVX recipients experienced 1.4 symptoms versus 2.2 in mRNA recipients (Fig 3). 15.7% of NVX recipients experienced at least one symptom of Grade 2 or higher versus 33.6% of mRNA recipients. NVX recipients had much fewer grade 2 or 3 reactogenicity events than mRNA. An absolute difference of 11.7% fewer NVX versus mRNA recipients reported solicited local reactogenicity events. The occurrence and severity of the solicited reactogenic events were transient with no difference reported at 6 days post-vaccination (Fig 2).

**Conclusion:**

The lower frequency and intensity of COVID-19 reactogenicity symptoms observed in this trial suggests use of the 2023/2024 adjuvanted NVX vaccine as an immunization option with lower reactogenicity. These findings may provide insight for policymakers and clinicians regarding reactogenicity that may influence patient behavior and impact choice of COVID-19 vaccination.

**Disclosures:**

Sarang K. Yoon, DO, MOH, Novavax: Grant/Research Support Andrew Phillips, MD, MOH, Novavax: Grant/Research Support German L. Ellsworth, MD, MPH, MOH, Novavax: Grant/Research Support Sarah W. Ball, MPH, ScD, Centers for Disease Control and Prevention, Contract #200-2019-F-06819: Grant/Research Support|Centers for Disease Control and Prevention, Contract #75D30121D12779: Grant/Research Support|Novavax: Grant/Research Support Elizabeth Rowley, DrPH, Centers for Disease Control and Prevention: Grant/Research Support|Novavax: Payments made to Westat, Inc. (employer)|University of Utah: Funded under contract with the University of Utah with prime funding and drug provided by Novavax Steph Battan-Wraith, PhD, Novavax, Inc.: Grant/Research Support Rebecca Fink, MPH, Novavax, Inc.: Grant/Research Support Adam Yates, PhD, Beehive Study: Grant/Research Support|Centers for Disease Control and Prevention, Contract #200-2019-F-06819: Grant/Research Support Seth Toback, M.D., Novavax, Inc.: employee|Novavax, Inc.: Stocks/Bonds (Public Company) Lisa M. Dunkle, MD, Novavax, Inc.: contractor for Novavax|Novavax, Inc.: Stocks/Bonds (Public Company) Matthew D. Rousculp, PhD, Novavax, Inc.: employee|Novavax, Inc.: employee|Novavax, Inc.: Stocks/Bonds (Private Company)|Novavax, Inc.: Stocks/Bonds (Private Company)

